# Deep Learning–Assisted Burn Wound Diagnosis: Diagnostic Model Development Study

**DOI:** 10.2196/22798

**Published:** 2021-12-02

**Authors:** Che Wei Chang, Feipei Lai, Mesakh Christian, Yu Chun Chen, Ching Hsu, Yo Shen Chen, Dun Hao Chang, Tyng Luen Roan, Yen Che Yu

**Affiliations:** 1 Graduate Institute of Biomedical Electronics & Bioinformatics National Taiwan University Taipei Taiwan; 2 Division of Plastic and Reconstructive Surgery Department of Surgery Far Eastern Memorial Hospital New Taipei Taiwan; 3 Department of Computer Science & Information Engineering National Taiwan University Taipei Taiwan; 4 Department of Information Management Yuan Ze University Chung-Li Taiwan

**Keywords:** deep learning, semantic segmentation, instance segmentation, burn wounds, percentage total body surface area

## Abstract

**Background:**

Accurate assessment of the percentage total body surface area (%TBSA) of burn wounds is crucial in the management of burn patients. The resuscitation fluid and nutritional needs of burn patients, their need for intensive unit care, and probability of mortality are all directly related to %TBSA. It is difficult to estimate a burn area of irregular shape by inspection. Many articles have reported discrepancies in estimating %TBSA by different doctors.

**Objective:**

We propose a method, based on deep learning, for burn wound detection, segmentation, and calculation of %TBSA on a pixel-to-pixel basis.

**Methods:**

A 2-step procedure was used to convert burn wound diagnosis into %TBSA. In the first step, images of burn wounds were collected from medical records and labeled by burn surgeons, and the data set was then input into 2 deep learning architectures, U-Net and Mask R-CNN, each configured with 2 different backbones, to segment the burn wounds. In the second step, we collected and labeled images of hands to create another data set, which was also input into U-Net and Mask R-CNN to segment the hands. The %TBSA of burn wounds was then calculated by comparing the pixels of mask areas on images of the burn wound and hand of the same patient according to the rule of hand, which states that one’s hand accounts for 0.8% of TBSA.

**Results:**

A total of 2591 images of burn wounds were collected and labeled to form the burn wound data set. The data set was randomly split into training, validation, and testing sets in a ratio of 8:1:1. Four hundred images of volar hands were collected and labeled to form the hand data set, which was also split into 3 sets using the same method. For the images of burn wounds, Mask R-CNN with ResNet101 had the best segmentation result with a Dice coefficient (DC) of 0.9496, while U-Net with ResNet101 had a DC of 0.8545. For the hand images, U-Net and Mask R-CNN had similar performance with DC values of 0.9920 and 0.9910, respectively. Lastly, we conducted a test diagnosis in a burn patient. Mask R-CNN with ResNet101 had on average less deviation (0.115% TBSA) from the ground truth than burn surgeons.

**Conclusions:**

This is one of the first studies to diagnose all depths of burn wounds and convert the segmentation results into %TBSA using different deep learning models. We aimed to assist medical staff in estimating burn size more accurately, thereby helping to provide precise care to burn victims.

## Introduction

### Background

According to the World Health Organization, an estimated 265,000 deaths occur each year from burn injuries. In the United States, burn injuries result in 10 million visits to the emergency department and 40,000 patients requiring hospitalization annually. The most critical aspect of managing burn injuries is the accurate calculation of the burn area, expressed as percentage total body surface area (%TBSA). However, many articles have reported discrepancies in the %TBSA diagnosed by different doctors. In adult burn injuries, Harish et al reported that overestimation by the referring institution occurred in 53% of cases and that the difference was statistically significant [1]. In child burn injuries from a national survey, Baartmans et al reported that burn size was often overestimated by referrers, by up to 30% TBSA, while underestimation was up to 13% TBSA [2].

There are 2 types of inaccurate estimations of burn injuries: misdiagnosis of burn depth and miscalculation of burn area. Misdiagnosis of burn depth comes from the dynamic nature of wound change. The initial presentation of burn depth may be quite different from the presentation several days after injury. Hence, the reported accuracy of diagnosis of burn depth is only 64% to 76% among experienced burn surgeons [3]. When evaluations are performed by less experienced practitioners, the accuracy declines to 50%. Fortunately, many technologies have been developed for accurate diagnosis of burn depth, such as laser Doppler imaging (LDI), infrared thermography, and photoacoustic imaging [4-7]. For example, LDI, which is based on perfusion in the burn area, provides information that is highly correlated with burn wound healing potential. Healing potential is a practical indicator of burn depth.

Though the assessment of burn depth with such technologies is often satisfactory, miscalculation of burn area may be hard to avoid. Such miscalculation often occurs when an area of irregular shape is estimated by comparing it with another area of irregular shape, for example, estimating the %TBSA of an irregularly shaped burn area on the upper extremity of an adult using the estimation that the upper extremity has roughly 7% to 9% TBSA as a guide [8,9]. In an interesting study, Parvizi et al reported that even when participants reached consensus on the margin of the burn wound, their estimations of %TBSA were still different [10]. The difference in %TBSA resulted in discrepancies in estimating the amount of resuscitation fluid needed by as much as 5280 mL using the Parkland formula. Clearly, there is an unmet need to improve the accuracy of burn diagnosis.

Machine learning has many applications in the field of medicine, such as in drug development and disease diagnosis [11-14]. Although machine learning has also been implemented in many aspects of surgery, its application in burn care is relatively rare [15,16]. Burn care is a field where human error can be reduced by computer assistance.

### Prior Work

Early work in the use of machine learning to assist burn diagnosis focused on classification of burn depth (Table 1). Since burn injuries result in a mixture of different burn depths, most images of burn wounds cannot be simply classified as superficial partial burn, deep partial burn, or full thickness burn. Before images of burn wounds are input for feature extraction, the images need to be processed.

**Table 1:** Segmentation of burn wounds.

Study	Image database	Model	Performance metric	Objective
Serrano et al [17]	38 images	Fuzzy-ARTMAP	Accuracy 88.57%	Burn depth
Acha et al [18]	50 images	Fuzzy-ARTMAP	Accuracy 82.26%	Burn depth
Acha et al [19]	50 images	SVM^a^, Fuzzy-ARTMAP	Error rate 0.7%	Burn depth
Acha et al [20]	74 images	KNN^b^, MDS^c^	Accuracy 83.8%	Need for skin grafts
Serrano et al [21]	94 images	SVM, MDS	Accuracy 79.73%	Need for skin grafts
Cirillo et al [22]	23 images	VGG16, GoogleNet, ResNet50, ResNet101	Accuracy 90.54%	Burn depth
Despo et al [23]	749 images	AlexNet, VGG16, GoogleNet	Accuracy 85%	Burn area segmentation, burn depth
Jiao et al [24]	1000 images	Mask R-CNN	DC^d^ 84.51%	Burn area segmentation
Our study	2591 images	Mask R-CNN, U-Net	DC 94%	Estimation of burn %TBSA^e^

^a^SVM: support vector machine.

^b^KNN: K-nearest neighbor.

^c^MDS: multidimensional scaling.

^d^DC: Dice coefficient.

^e^%TBSA: percentage total body surface area.

#### Small Regions of Images

The most common method of addressing different burn depths in a given image is to select small regions of the image, called boxes, for processing. These small boxes are then transformed into a red/green/blue (RGB) matrix in a color coordinate system. The relative distance of each of the pixels from the others is then calculated and a threshold is set to check whether the box is homogeneous in texture and color. Homogeneous boxes are classified into different burn depths and input for machine learning.

Acha and Serrano collected 62 images of burn wounds with a resolution of 1536×1024 pixels. They selected regions of only 49×49 pixels from the images and classified these small boxes into 5 appearances to yield 250 images. They input the data set into Fuzzy-ARTMAP for training. A neural network was then used to classify burns into the 3 aforementioned types of burn depths with a success rate of 82% to 88% [17,18]. Later, they reduced the error rate from 1.6% to 0.7% by applying 5-fold cross-validation to the data sets and used support vector machine (SVM) to perform the classification [19]. In 2 subsequent studies, they further applied multidimensional scaling combining SVM and k-nearest neighbor classification to predict the need for a skin graft, with success rates of 79.73% and 83.8%, respectively [20,21].

#### Continuous Monitoring

Another method used to get the burn depths of a region corresponding to any specified pixels of the images of a burn wound is to record the wound from the time of injury to complete healing with the same protocol. Cirillo et al continuously collected images from the same burn wound until it healed [22]. They were then able to draw lines on the image corresponding to healing time and divide the area into 4 types of burn depths. To be more precise, they used the method mentioned above to extract small regions of the images (676 regions of 224×224 pixels from 23 images of 3456×2304 pixels). They then input these square regions of interest (RoIs) into several pretrained convolutional neural network (CNN) models, such as VGG19, ResNet18, ResNet50, and ResNet101. ResNet101 showed the best classification results with an average accuracy of 0.8166.

### Goal of This Study

The use of machine learning in burn diagnosis to classify burn depth is currently quite limited. Technologies, such as LDI and thermography, are readily available and far more commonly employed. The treatment of burn injury may last for days or months. Without the use of special technologies, burn depth can still be determined by clinical assessment during the course of treatment. Recently, CNNs have been used in burn diagnosis to segment burn wounds. Despo et al reported a mean intersection over union (IoU) of around 0.7 with a fully convolutional network (FCN) [23]. Jiao et al reported a mean Dice coefficient (DC) of 0.85 with Mask R-CNN [24]. Such segmentation results could further be used to calculate %TBSA. This is important because all formulae for emergent fluid resuscitation (eg, the Parkland formula = %TBSA × body weight × 4) and calorie needs (eg, the Curreri formula = 25 × body weight + 40 × %TBSA) are based on %TBSA.

In this study, we implemented deep learning models to segment burn wounds and perform conversion to %TBSA based on the number of pixels. We tried to decrease the human error of estimating an area of irregular shape by inspection. We aimed to help medical staff obtain accurate formulae to aid in making decisions about triage, acute management, and transfer of burn patients.

## Methods

### Image Acquisition

This study was approved by the research ethics review committee of Far Eastern Hospital (number 109037-F). We reviewed the medical records of patients in Far Eastern Hospital from January 2016 to December 2019 with ICD9 codes 940-948, 983, and 994. We collected the images of burn wounds from their medical records and saved them as JPG files. These images were assigned random numbers for deidentification and were randomly presented to 2 out of 5 burn surgeons for labeling.

### Labeling and Processing

Since many burn wounds have a mixture of different burn depths, the images were roughly classified into the following 3 categories: superficial/superficial partial burn, deep partial burn, and full thickness burn. Clinically, the color of superficial/superficial partial burns is red or pink, and the color of deep partial burns is dark pink to blotchy red. Blistering is common in superficial partial burns and is also present in deep partial burns of a relatively large size. Full thickness burns are white, waxy, or charred without blisters. All images were co-labeled by 2 burn surgeons to yield a single consensus result. The margins of the burn wounds were labeled without regard to burn depth with the labeling tool *LabelMe* and saved as JSON files. A burn wound image was excluded if the wound was on the face; it involved tattooed skin; it was coated with burn ointment; it appeared to have undergone an intervention, such as debridement or skin graft; or no agreement was reached on the margin of the burn wound by the 2 burn surgeons.

Since the images of burn wounds were collected from various medical records, their sizes were not uniform and ranged from 4000×3000 to 2736×1824 to 2592×1944 pixels. All labeled images were resized to 512×512 pixels. The data set of burn wounds was randomly split in a ratio of 8:1:1 into 3 sets for training, validation, and testing. We applied 2 deep learning architectures, U-Net and Mask R-CNN, in combination with 2 different backbones, ResNet50 and ResNet101, to segment these images.

### Evaluation Metrics

The DC and IoU are 2 common metrics used to assess segmentation performance, whereas precision, recall, and accuracy are common metrics for assessing classification performance. The DC is twice the area of the intersection of the ground truth and prediction divided by the sum of their areas. It is given as follows:




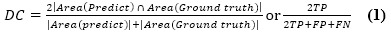




where TP (true positive) denotes the number of correctly classified burn pixels, FP (false positive) denotes the number of mistakenly classified burn pixels, and FN (false negative) denotes the number of mistakenly classified nonburn pixels.

The IoU denotes the area of the intersection of the ground truth and prediction divided by the area of their union. It is given as follows:




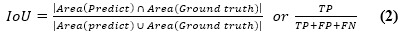




Precision is defined as the ratio of burn pixels that models correctly classified in all predicted pixels. It is also called positive predictive value and is given as follows:

Precision = TP / (TP + FP) **(3)**

Recall is defined as the ratio of burn pixels that are correctly classified in all actual burn pixels. It is also called sensitivity and is given as follows:

Recall = TP / (TP + FN) **(4)**

Accuracy denotes the percentage of correctly classified pixels. It is given as follows:

Accuracy = (TP + TN) / (TP + FP + TN + FN) **(5)**

where TN (true negative) denotes the number of correctly classified nonburn pixels.

### Semantic Segmentation: U-Net

The convolutions in the U-Net path can be replaced with a deep network framework, such as the ResNet framework, which can explore and learn more features from the data (Multimedia Appendix 1). Then, the networks can be initialized using pretrained model weights derived from large-scale object detection, segmentation, and captioning data sets such as ImageNet and COCO. In our case, we trained our model using 2 different backbones, ResNet101 and ResNet50, with weights from the pretrained ImageNet model (Table 2). The standard augmentations of images we used were rotations, shifts, scale, gaussian blur, and contrast normalization. The standard Dice loss was chosen as the loss function. The formula is as follows:




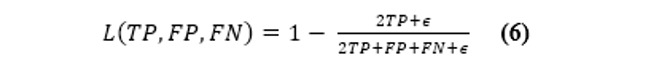




The *ϵ* term is used to avoid the issue of dividing by 0 when precision and recall are empty.

**Table 2:** Configuration of the models.

Variable	Mask R-CNN	U-Net
Number of classes	1	1
Backbone	ResNet101 & ResNet50	ResNet101 & ResNet50
Regional proposal network anchor scales	8, 16, 32, 64, 128	N/A^a^
Train RoIs^b^ per image, n	128	N/A
Anchors per image, n	256	N/A
Learning rate	0.0001 (initial rate, change in different epochs)	0.001
Learning momentum	0.9	0.9
Weight decay	0.0001	N/A
Batch size	8	8
Image dimensions	512×512	512×512

^a^N/A: not applicable.

^b^RoI: region of interest.

### Instance Segmentation: Mask R-CNN

**Figure 1:**
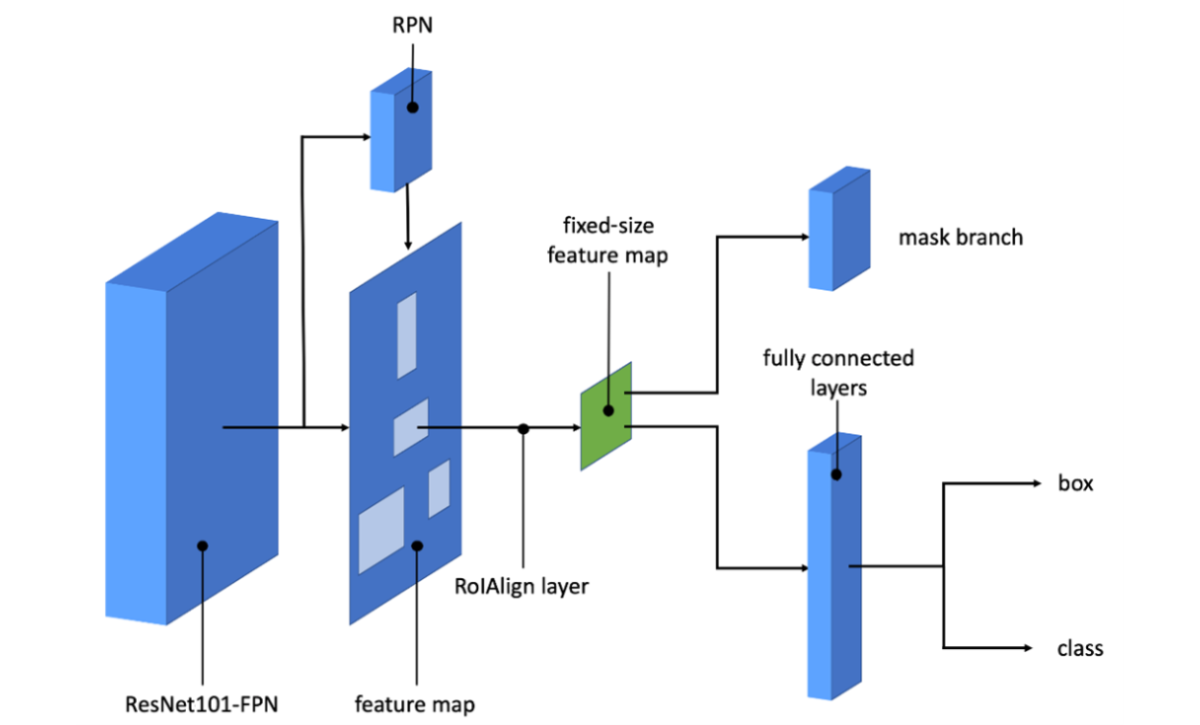
Mask R-CNN architecture with ResNet101. FPN: feature pyramid network; RoI: region of interest; RPN: regional proposal network.

In our implementation of Mask R-CNN, we trained our model using ResNet101 and ResNet50 with weights from the pretrained COCO model (Table 1). Mask R-CNN uses a multitask loss function given by L = L_class_ + L_box_ + L_mask_ (Figure 1). The L_class_ component contains the regional proposal network (RPN) class loss (failure of the RPN to separate object prediction from background) added to the Mask R-CNN class loss (failure of Mask R-CNN object classification). The L_box_ component contains the RPN bounding box loss (failure of object localization or bounding by the RPN) added to the Mask R-CNN bounding box loss (failure of object localization or bounding by Mask R-CNN). The last component L_mask_ loss constitutes the failure of Mask R-CNN object mask segmentation.

### Burn Segmentation to %TBSA

When the burn wounds are correctly segmented, the final step is to convert the pixels to %TBSA. To solve this problem, we applied the rule of hand/palm. The original rule is that a person’s hand with digits accounts for 1% TBSA. It is the most common method of estimating burn %TBSA [25,26]. Recent studies have shown that a hand without digits represents precisely 0.5% TBSA (the rule of palm) and a hand with digits should be adjusted to around 0.8% TBSA (the rule of hand) [8]. If we use deep learning models to segment a patient’s burn wounds as well as hands, we can then convert the segmentation result of burn wounds into %TBSA.

To produce the data set of hands and the data set of palms, we collected images of both volar hands from our colleagues. For each image, we labeled the hand with digits and without digits corresponding to the rule of hand and the rule of palm, respectively. These 2 data sets were split in a ratio of 8:1:1 into training, validation, and testing sets as well. The hand data set and the palm data set were processed according to the previous methods for burn wounds. The %TBSA of a burn wound can be calculated by comparing the mask area of the burn wound with the mask area of the hand or palm of the same patient. The formula is given by:




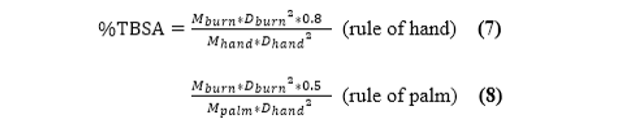




where M_burn_ is the number of pixels of the masked burn area, M_hand_ is the number of pixels of the masked hand area (0.8% TBSA), M_palm_ is the number of pixels of the masked palm area (0.5% TBSA), D_burn_ is the filming distance of the image of the patient’s burn wound, and D_hand_ is the filming distance of the image of the patient’s hand.

## Results

### Segmentation of Burn Wounds

There were 3 data sets used in our study, 1 each for burn wounds, hands, and palms. For the burn wound data, we collected 3571 images from the medical records of Far Eastern Hospital, 980 of which were excluded (mostly because the burn wounds had undergone interventions, and some because they were coated with burn ointment). The 2591 selected images were labeled and included in the burn wound data set. Among these images, 2073 were used as the training set and 259 were used as the validation set. The remaining 259 images were preserved as the testing set.

In our study, there was only 1 class in the ground truth. From the definitions of the DC and IoU, they have the relation of 1/2 × DC ≤ IoU ≤ DC and perfect positive correlation. We used DC as our main metric to evaluate segmentation performance because it penalizes false negatives more than IoU does, and it is better to overestimate burn size than underestimate it.

Both U-Net and Mask R-CNN had better segmentation performance with the ResNet101 backbone than with ResNet50 (Table 3 and Table 4). The improvement was obvious in U-Net (DC: 0.8545 vs 0.8077) but negligible in Mask R-CNN (DC: 0.9496 vs 0.9493). Under the same backbone, Mask R-CNN had better performance in burn wound segmentation and classification than U-Net. Mask R-CNN with ResNet101 had the best segmentation result with a DC of 0.9496.

**Figure 2:**
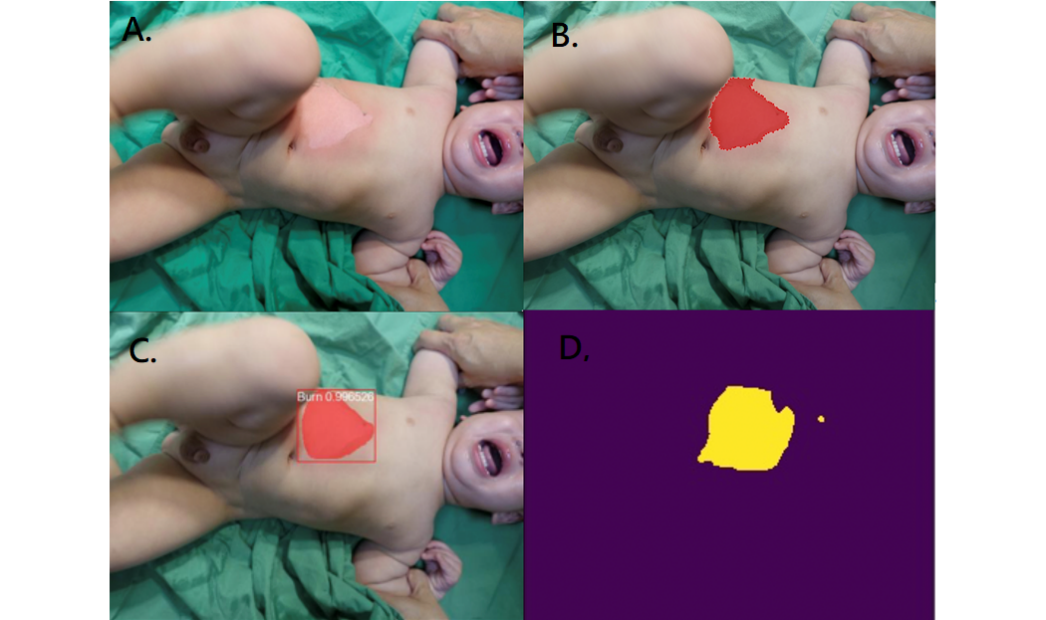
Superficial partial burn. A: original photo; B: ground truth; C: result of Mask R-CNN; D: result of U-Net.

**Figure 3:**
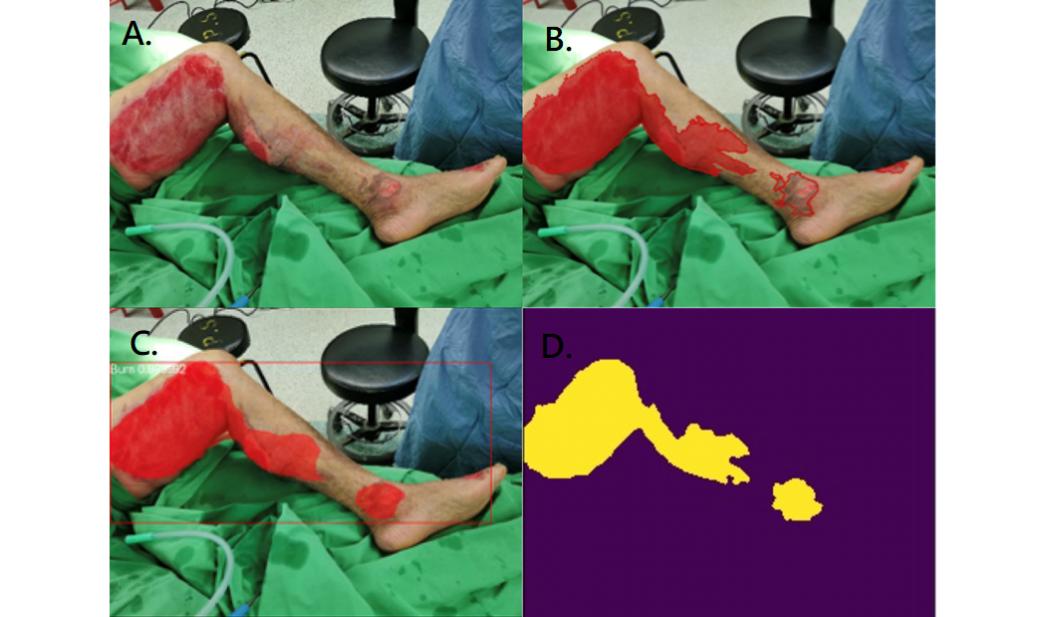
Deep partial burn. A: original photo; B: ground truth; C: result of Mask R-CNN; D: result of U-Net.

**Figure 4:**
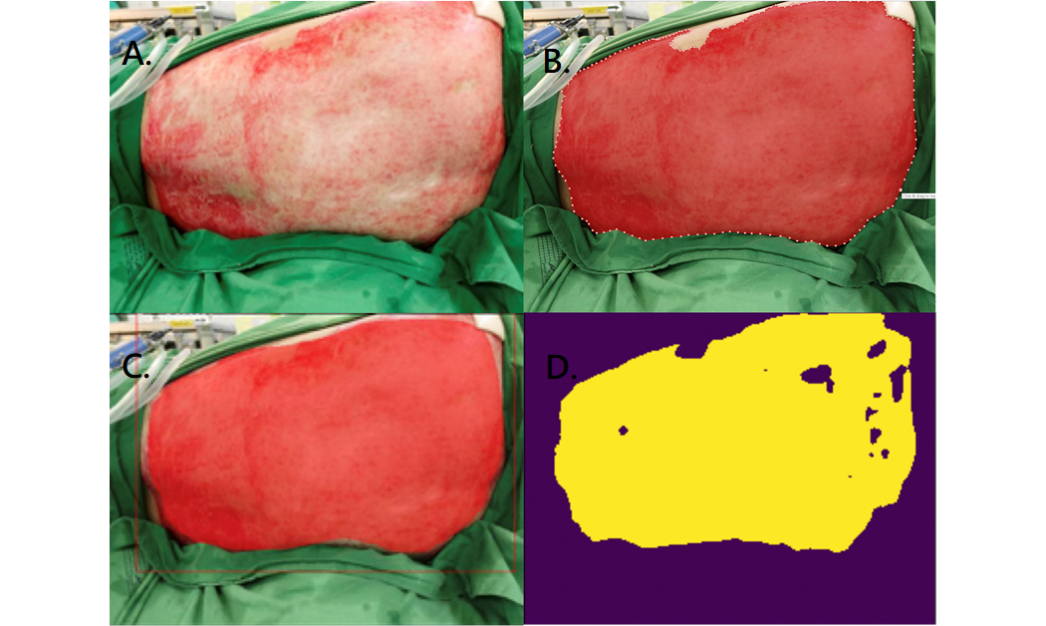
Full thickness burn. A: original photo; B: ground truth; C: result of Mask R-CNN; D: result of U-Net.

**Figure 5:**
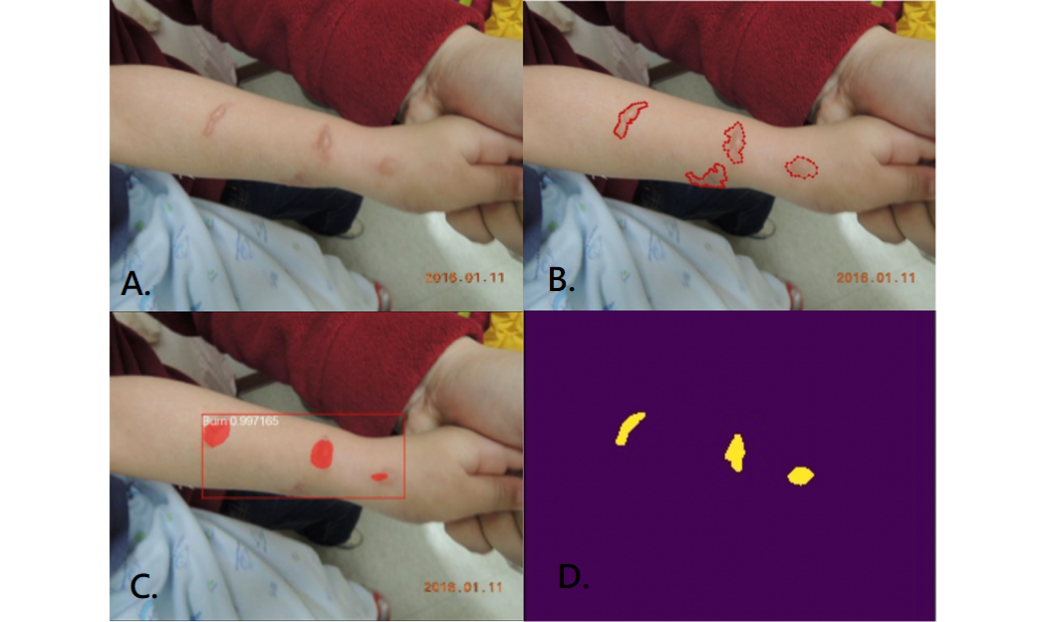
Small scattered burns. A: original photo; B: ground truth; C: result of Mask R-CNN; D: result of U-Net.

Figures 2-4 illustrate the performance of the 2 models in segmenting different burn depths. Both Mask R-CNN and U-Net showed poor segmentation results when they encountered small scattered burns (Figure 5).

**Table 3:** Segmentation results of burn wounds with ResNet101.

Variable	U-Net	Mask R-CNN
Mean DC^a^	0.8545	0.9496
Mean IoU^b^	0.7782	0.9089
Mean precision	0.9041	0.9613
Mean recall	0.8541	0.9390
Mean accuracy	0.7893	0.9130

^a^DC: Dice coefficient.

^b^IoU: intersection over union.

**Table 4:** Segmentation results of burn wounds with ResNet50.

Variable	U-Net	Mask R-CNN
Mean DC^a^	0.8077	0.9493
Mean IoU^b^	0.7190	0.9075
Mean precision	0.8947	0.9610
Mean recall	0.8002	0.9382
Mean accuracy	0.7331	0.9117

^a^DC: Dice coefficient.

^b^IoU: intersection over union.

### Segmentation of Hands and Palms

A total of 400 images of both volar hands were collected and labeled. The male-to-female ratio was 193:207. Since U-Net and Mask R-CNN both performed better with the ResNet101 backbone than with the ResNet50 backbone in the burn wound segmentation, only ResNet101 was applied in the segmentation of the hand and palm data sets.

**Figure 6:**
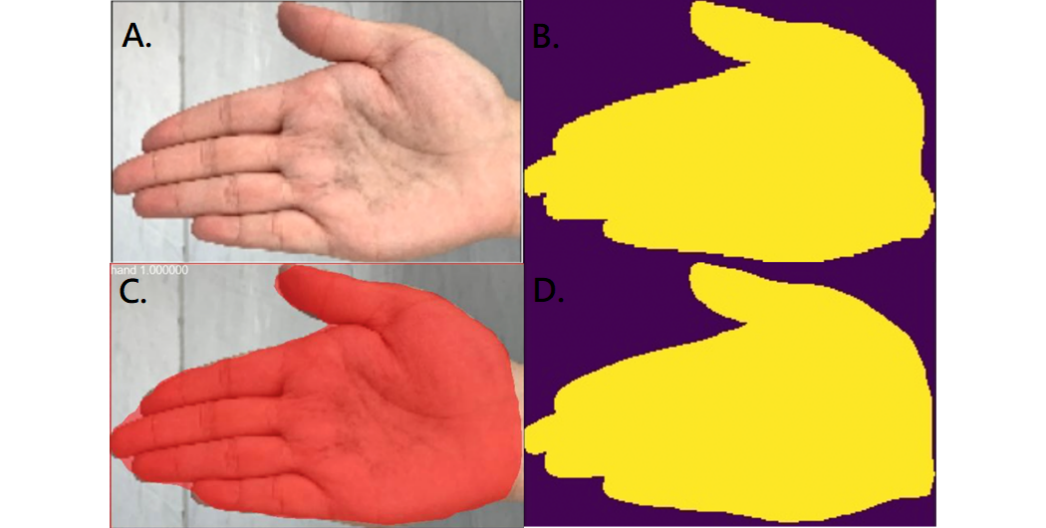
Segmentation of the hand. A: original photo; B: ground truth; C: result of Mask R-CNN; D: result of U-Net.

Contrary to the burn wound results, U-Net had slightly better overall performance in the segmentation of the hands and palms than Mask R-CNN (Table 5 and Table 6). For hand segmentation, U-Net had a DC of 0.9920 and Mask R-CNN had a DC of 0.9692. For palm segmentation, the difference was not as obvious with a DC of 0.9910 versus 0.9803. Figure 6 provides a representative example of the segmentation of a particular hand by both U-Net and Mask R-CNN, while Multimedia Appendix 2 provides an example for a palm.

**Table 5:** Segmentation results for hands with ResNet101.

Variable	U-Net	Mask R-CNN
Mean DC^a^	0.9920	0.9692
Mean IoU^b^	0.9842	0.9405
Mean precision	0.9906	0.9657
Mean recall	0.9935	0.9728
Mean accuracy	0.9933	0.9407

^a^DC: Dice coefficient.

^b^IoU: intersection over union.

**Table 6:** Segmentation results for palms with ResNet101.

Variable	U-Net	Mask R-CNN
Mean DC^a^	0.9910	0.9803
Mean IoU^b^	0.9822	0.9614
Mean precision	0.9904	0.9836
Mean recall	0.9916	0.9770
Mean accuracy	0.9878	0.9615

^a^DC: Dice coefficient.

^b^IoU: intersection over union.

### Burn Segmentation to %TBSA

**Figure 7:**
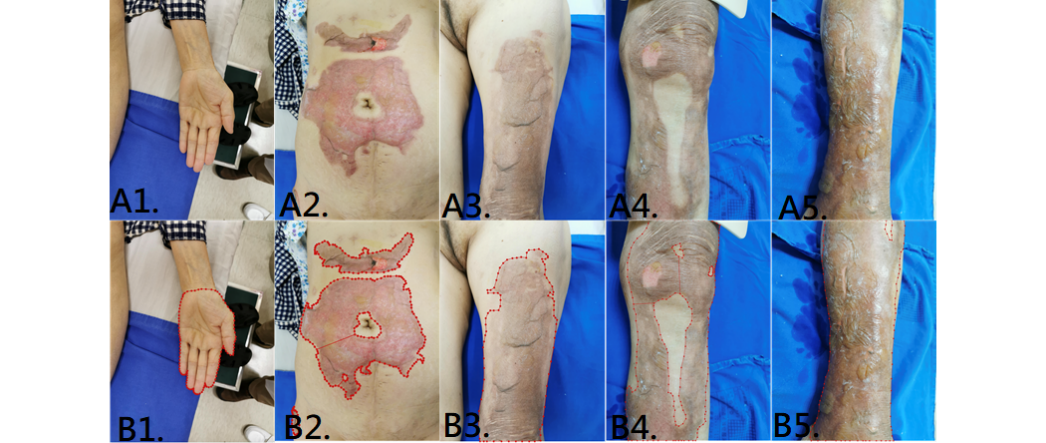
A1-A5: original image of the left hand, abdomen, left thigh, right leg, and left leg. B1-B5: labeled images as ground truth.

**Figure 8:**
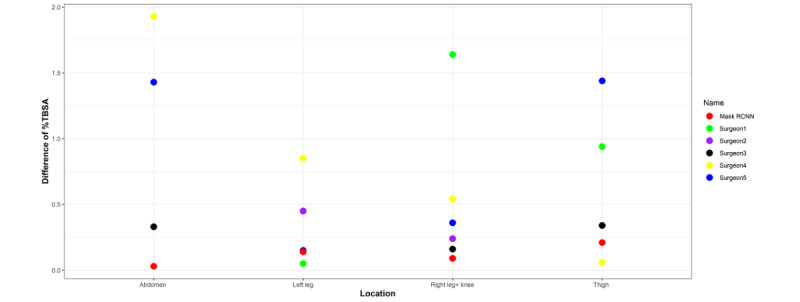
Differences between ground truth and estimated %TBSA of Mask R-CNN and burn surgeons at various burn sites. %TBSA: percentage total body surface area.

In the last part of our study, we designed a test to compare the estimation of the percentage of TBSA burned according to surgeons and Mask R-CNN. Photos of the abdomen, left thigh, left leg, right leg, and left hand of a patient were taken from the same distance (Figure 7). Images of the burn wounds and of the hands were co-labeled by 2 surgeons as ground truth. The previously trained Mask R-CNN with the ResNet101 backbone was used to calculate the %TBSA of each wound. Then, pictures of the burn wounds and the hands were given to 5 burn surgeons, and they gave their respective estimations of %TBSA. The results of each surgeon, ground truth, and Mask R-CNN are shown in Multimedia Appendix 3. The ground truth was a pixel-based calculation (abdomen: 2.07%, thigh: 2.06%, right leg and knee: 2.64%, and left leg: 2.85%). Mask R-CNN had a smaller average deviation (0.115% TBSA) from ground truth than all of the burn surgeons (0.45%-1.14% TBSA; Figure 8).

## Discussion

### Data Sets

Studies of machine learning in burn diagnosis are relatively rare, because there are challenges in establishing accurate data sets. To begin with, unlike medical images from X-ray or computed tomography (CT) scans, images of burn wounds are not acquired under a standard protocol. Images of burn wounds are acquired using different equipment under various circumstances, such as illumination conditions, distance to the patient, and the background scene. These factors make it difficult to achieve a uniform standard of labeling and annotation.

Next, the numbers of burn images compared with other open image data sets, such as MNIST (70,000 images) and CIFAR-10 (60,000 images), are limited. In recent studies of burn wound segmentation, Despo et al used 656 images for training [23] and Jiao used 1000 images for training [24]. We used 2332 labeled images from all burn depths for training and 259 images for testing. Images of burn wounds are difficult to collect. Unlike cancer imaging archives, there are no high-quality open data sets of images of burn wounds. This may be because complete deidentification of these images is not possible. Researchers are asked not to publish these images as open data sets due to patient privacy. Researchers from different medical facilities are not permitted to share the images with each other as well. Under these circumstances, federated learning to form a global model may be a feasible method to improve the accuracy of different individual models. The concept of federated learning is to share only the weights and bias of different models without sharing data sets [27,28].

In addition, burn wounds, unlike tumors that are detected on magnetic resonance imaging (MRI) images, are not commonly sampled for biopsy to confirm diagnosis. For any pixels on the images, if no other diagnostic technology is used, the true burn depths are hard to ascertain. The images, even when labeled by burn specialists, are relative ground truth only. A given image may receive many different labels when assessed by many doctors.

Finally, many burn wounds have a mixture of several burn depths. If the object of deep learning is to build a burn depth classifier, most images cannot be included for training. Images of burn wounds require preprocessing as discussed previously in the methods.

In the early work of our study, we tried to build a burn depth classifier. We divided the images of burn wounds into the following 4 categories based on burn depth: superficial (112 images), superficial partial (201 images), deep partial (165 images), and full thickness (170 images). We imported the data set into IBM Visual Insights (previously PowerAI Vision), a tool that can train models to do the classification task. We did data augmentation to enlarge the data set and improve generalization. Then, we chose pretrained GoogLeNet as our network structure. This model showed decent results, with a mean accuracy of 93% (Multimedia Appendix 4). However, some images in the category “superficial partial” had regions with other burn depths as well. The confusion matrix showed more false negative results in this group than in the others (Multimedia Appendix 5). Hence, the accuracy of the model as a burn depth classifier largely depended on the burn wound images collected.

The abovementioned confounding factors also had an impact in previous studies of machine learning used to segment images of burn wounds. In the study by Despo et al, the margins of burn wounds on images were labeled by a surgeon. Then, every image was annotated to 1 severity of burn depth. Since the burn wound depths were not homogeneous, accuracy and IoU were greater in partial thickness burns [23]. In our study, we also faced the same challenges. Initially, every image was labeled by 2 burn surgeons to obtain 2 labeled images. When the burn wounds had multiple burn depths, the labeled areas of the 2 surgeons had more discrepancy. When we input the discrepantly labeled images to train the models, they resulted in a good mask of the overall burn area but an incorrect classification of burn depth segmentation (Multimedia Appendix 6 and Multimedia Appendix 7). Zhang et al reported an interesting finding [29]. When they input randomly labeled objects or random pixels, after 10,000 steps, their neural network models still converged to fit the training set perfectly. The neural networks were rich enough to memorize bad training data. Yet, their results on testing data sets were poor. To avoid the problem of ambiguous ground truth, we modified the method so that only the burn wound margin was co-labeled by the 2 burn surgeons. This was because the ground truth of the margins had the highest consensus and because all formulae used for burn resuscitation only involved total burn area, which is equivalent to burn margin and is not related to burn depth.

### Segmentation Results

We chose U-Net and Mask R-CNN as our main models for segmentation of burn wounds and hands because they are both popular and well-developed CNN models. Although they have different architectures and use different loss functions, their segmentation output seems similar. U-Net outputs semantic segmentation, and it is the most common segmentation model in the medical field [30]. U-Net has been deployed in the evaluation of various sources of medical images, such as positron emission tomography (PET) scans of brain lesions [31], microscopy images of cells [32], CT scans of thoracic organs [33], and MRI scans of breast lesions [34]. Mask R-CNN was developed by Facebook AI Research, and it outputs object detection with instance segmentation [35,36]. Mask R-CNN began getting attention in the medical field in 2018. It has been deployed in the analysis of various sources of medical images as well, such as PET scans of lung lesions [37], sonographic images of breast lesions [38], and MRI scans of knee injuries [39].

Previous studies have also applied these 2 models. Vuola et al reported a study of nuclei segmentation of microscopy images. U-Net had a better DC and created more accurate segmentation masks. Mask R-CNN had better recall and precision, and could detect nuclei more accurately but struggled to predict a good segmentation mask [40]. Zhao et al reported a study of tree canopy segmentation of aerial images. Mask R-CNN performed better in segmentation as well as in tree detection [41]. Bouget et al reported a study of thoracic structure segmentation combining 2 models. Mask R-CNN had the weakness of underestimating structural boundaries, and it required a longer training time. U-Net had the weakness of spatial inconsistency when compiling 2D segmentation results into 3D [42]. In our study, Mask R-CNN was better at burn wound segmentation, while U-Net was better at hand segmentation. We believe that when the segmented objects have similar shape and size, such as with nuclei, hands, and palms, U-Net can achieve better segmentation results than Mask R-CNN. Mask R-CNN had to take into account the loss function components from estimating the bounding box and class, not just the mask. The weights of the bounding box and class components are calculated prior to the weight of the mask component in order to get accurate instance location. Huang et al proposed a modified Mask R-CNN to improve mask prediction [43].

However, the performance of U-Net in burn wound segmentation was not as good as that of Mask R-CNN. The burn wounds comprised 3 types of burn depths with various colors, hues, and textures, and were also of irregular shape and different sizes. Because it lacks the RPN function of Mask R-CNN, U-Net may not have the volume to “memorize” all the features of burn wounds by convolution and de-convolution. In the Kaggle science bowl, both U-Net and Mask R-CNN achieved excellent results after fine tuning. Hence, the performance of the 2 models may depend on the segmentation task, the data sets, and fine tuning.

The segmentation result is not the only consideration. There are other comparative pros and cons of these 2 models. If a model is deployed in mobile devices, time consumption for prediction is an important factor. In our study, it took less time for U-Net (0.035 s/image) to do the prediction than for Mask R-CNN (0.175 s/image). The total time needed to train Mask R-CNN was about 1.5 times that needed to train U-Net. In addition, semantic segmentation involves direct pixel classification. If the objective is to calculate the total burn area, U-Net is capable of producing good results. If we want to segment different types of wounds on the same images, such as incisions and abrasions, Mask R-CNN can provide classification confidence in each of the RoIs, not just the masks.

Both U-Net and Mask R-CNN can segment burn wounds of any burn depths (Figures 2-4). The segmentation result was more satisfactory when areas were large and confluent (Figure 4). If the burn wound (pixels) was small, the segmentation results of both models were not satisfactory (Figure 5). This is because a small area is susceptible to resizing, convolution, and max pooling. Similar observations were reported by Bouget et al, when they segmented structures inside the chest wall [42]. Large structures, such as the heart, lungs, and spine, had a DC of more than 0.95. Small structures, such as lymph nodes, had a DC of only around 0.41. In the study by Vuola et al, they removed the very small masks (under 10 pixels) to improve the prediction [40]. Fortunately, small and scattered burns are less critical clinically.

### Conversion of Segmentation Mask to %TBSA

There exist other methods for converting a segmentation mask to %TBSA. One approach is to acquire the actual burn area (eg, 225 cm^2^) by calculating the relation of pixels of the mask area on the image and the distance from the wound to the camera. The next step is to calculate the body surface area (BSA; eg, 17,525 cm^2^) via the patient’s body weight, height, and gender. The %TBSA of the burn wound can be calculated by dividing these 2 numbers. Although this approach seems straightforward, there are more than 25 formulae to estimate BSA based on studies of different populations [44]. When it comes to child BSA, we need completely different formulae for calculation, again with various degrees of accuracy [45].

We adopted the rule of hand/palm as a guide to estimate %TBSA, because the rule of hand/palm shows very little difference between racial groups, genders, BMI, and ages [8,46]. The rule of hand/palm can also be used in children and infants, where it is closer to the original 1% TBSA rule. Moreover, thumbprints, which are approximately 1/30 TBSA, can also be used as a guide to estimate areas of small burns [47]. In our study, only 17 images were burn injuries involving the volar hand. We therefore collected images of healthy hands from our colleagues rather than using burned hands to train the models.

In the last stage of our study, we conducted a test to compare the %TBSA estimated by burn surgeons and by Mask R-CNN with a ResNet101 backbone. Mask R-CNN had less variance from ground truth on average. It is very important to have a small deviation on every estimation. If a patient has multiple burn sites, the errors from each wound may add up to become a large deviation. In a study by Parvizi et al, the difference in estimation by inspection across burn experts was found to be as large as 16.5% TBSA in an adult patient and 31.5% TBSA in a child patient, which resulted in great volume differences in the estimation of fluid needed for resuscitation [10]. Our method was aimed to derive similar estimates when the same burn wound was estimated by different burn experts by inspection, such as by teleconsultation. In reality, burn surgeons would typically visit patients and calculate the area more meticulously. Additionally, the burn area would be recalculated in the days following the burn injury. Theoretically, the variability among estimations would be less than when the burn area is estimated just by inspecting an image of the burn wound.

### Limitations

The data set of burn wounds was collected from a single medical center in Taiwan. Although it is currently the largest data set, the number of training images was small. The models require more input images to improve accuracy.

Our deep learning models can segment a burn wound of any burn depth. However, they are unable to classify burn depths on segmentation. This is so because the ground truth of burn depths is hard to define by burn surgeons consistently. Further study may apply machine learning to assist in burn depth labeling before input for training.

We used normal hands as a template to calculate the %TBSA burned. When a patient had burns involving both hands, our models could still segment the burned hands. Since children’s hands are shaped similarly to those of adults, our models can presumably also segment the hands of children (Multimedia Appendix 8). However, we did not collect enough images to directly assess accuracy in these circumstances.

Our data set did not include burn wounds from patients with markedly different skin tones. We hypothesize that the deep learning models will accurately detect burn wounds when the burn injury is more severe than superficial second degree, where the skin layers that are deeper than the pigment cells are disrupted. For example, a superficial second-degree burn injury with ruptured bullae shows a similar shade of pink even on different skin tones. Yet, skin tone will definitely contribute to the performance of the models. Convolution layers and the RoI obtained by deep learning largely depend on the relationship with their adjacent pixels. To test our hypothesis, we collected 100 web scraping images of burn wounds from different skin tones and input them into our models for wound segmentation (Multimedia Appendix 9). The results confirmed that our models performed well when the burn injury was more severe than superficial second degree. However, the segmentation results varied when the burn wound had no bullae formation or rupture (whether superficial second or first degree). To resolve this problem, we need more quality images to correlate skin tone with segmentation performance.

Finally, burn wound images are 2D projections of 3D burn wounds, akin to the Mercator world map. Unlike the world map, the cross sections of the trunk and extremities of the human body are not just ellipses or circles. The distance of the camera from the wound bed can be adjusted for by a simple formula, but adjusting for the angle at which the photos are taken requires complex differential and integral formulae with multiple variables. To get the most accurate estimation of %TBSA, we suggest taking all photos at a constant distance of around 30 to 50 cm and holding the camera (cellphone) parallel to the wound bed to decrease the effect of the angle. Our study will further deploy models on images taken with a 3D camera to acquire more accurate results.

### Conclusions

To the best of our knowledge, this is the first study to determine the %TBSA of burn wounds with different deep learning models. Based on the rule of hand, %TBSA can be calculated by comparing segmentation masks of the burn wound and hand of a patient. In our study, Mask R-CNN with ResNet101 performed this task satisfactorily in comparison with burn surgeons. With the assistance of deep learning, the fluid resuscitation and nutritional needs of burn injury patients can be more precisely and accurately assessed.

## Appendix

Multimedia Appendix 1 U-Net architecture, encoder, and decoder replaced with ResNet.

Multimedia Appendix 2 Segmentation of the palm. A: original photo; B: ground truth; C: result of Mask R-CNN; D: result of U-Net.

Multimedia Appendix 3 Estimation of %TBSA burned according to 5 different burn surgeons (ground truth and Mask R-CNN). %TBSA: percentage total body surface area.

Multimedia Appendix 4 Mean accuracy of burn depth classification.

Multimedia Appendix 5 Confusion matrix of different subgroups.

Multimedia Appendix 6 A mix of all burn depths in a burn wound.

Multimedia Appendix 7 Incorrect prediction of burn depths but correct prediction of total burn area.

Multimedia Appendix 8 A: adult hand burn; B: segmentation of adult hand burn; C: child hand burn; D: segmentation of child hand burn.

Multimedia Appendix 9 Web scraping images of burn wounds on patients with markedly lighter and darker skin tone in comparison to our study population.

## Abbreviations

%TBSA: percentage total body surface area
BSA: body surface area
CNN: convolutional neural network
CT: computed tomography
DC: Dice coefficient
IoU: intersection over union
LDI: laser Doppler imaging
MRI: magnetic resonance imaging
PET: positron emission tomography
RoI: region of interest
RPN: regional proposal network
SVM: support vector machine
