# Supplementary figures and images for: Deep Learning–Assisted Burn Wound Diagnosis: Diagnostic Model Development Study
Source: JMIR Med Inform. 2021 Dec 2;9(12):e22798. doi: 10.2196/22798 (PMC8686480; doi:10.2196/22798)

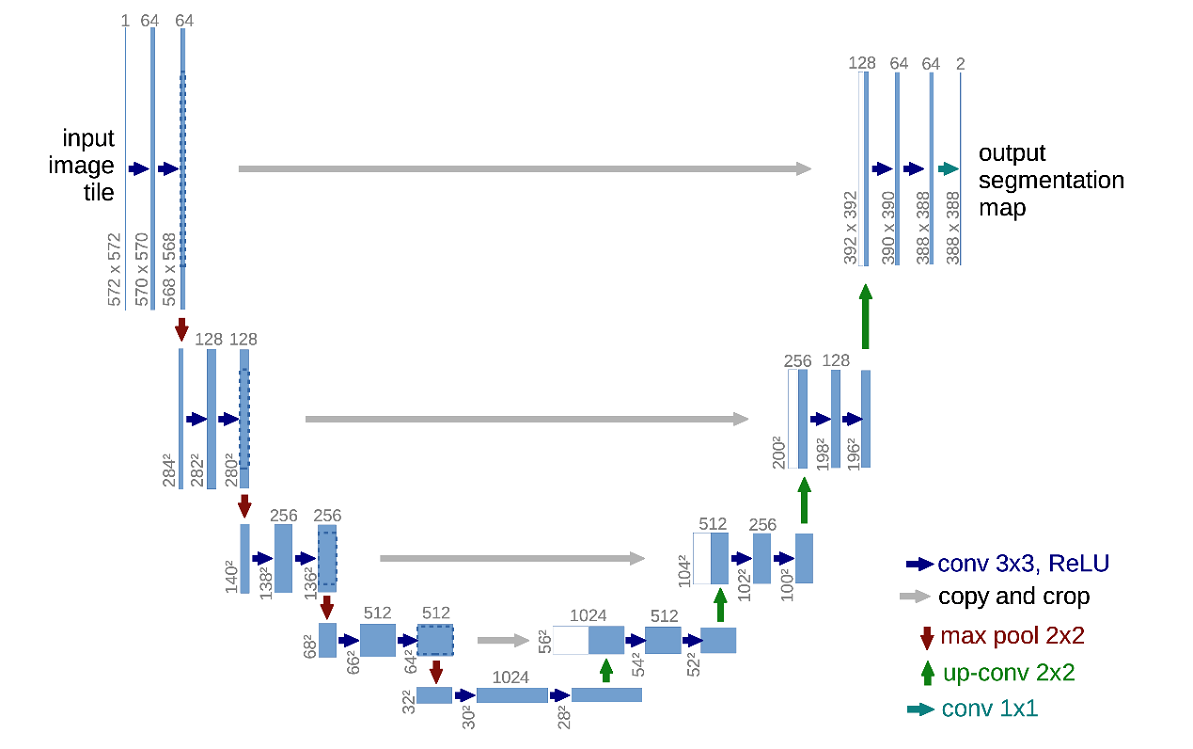

Supplement: Multimedia Appendix 1 [file medinform_v9i12e22798_app1.png]

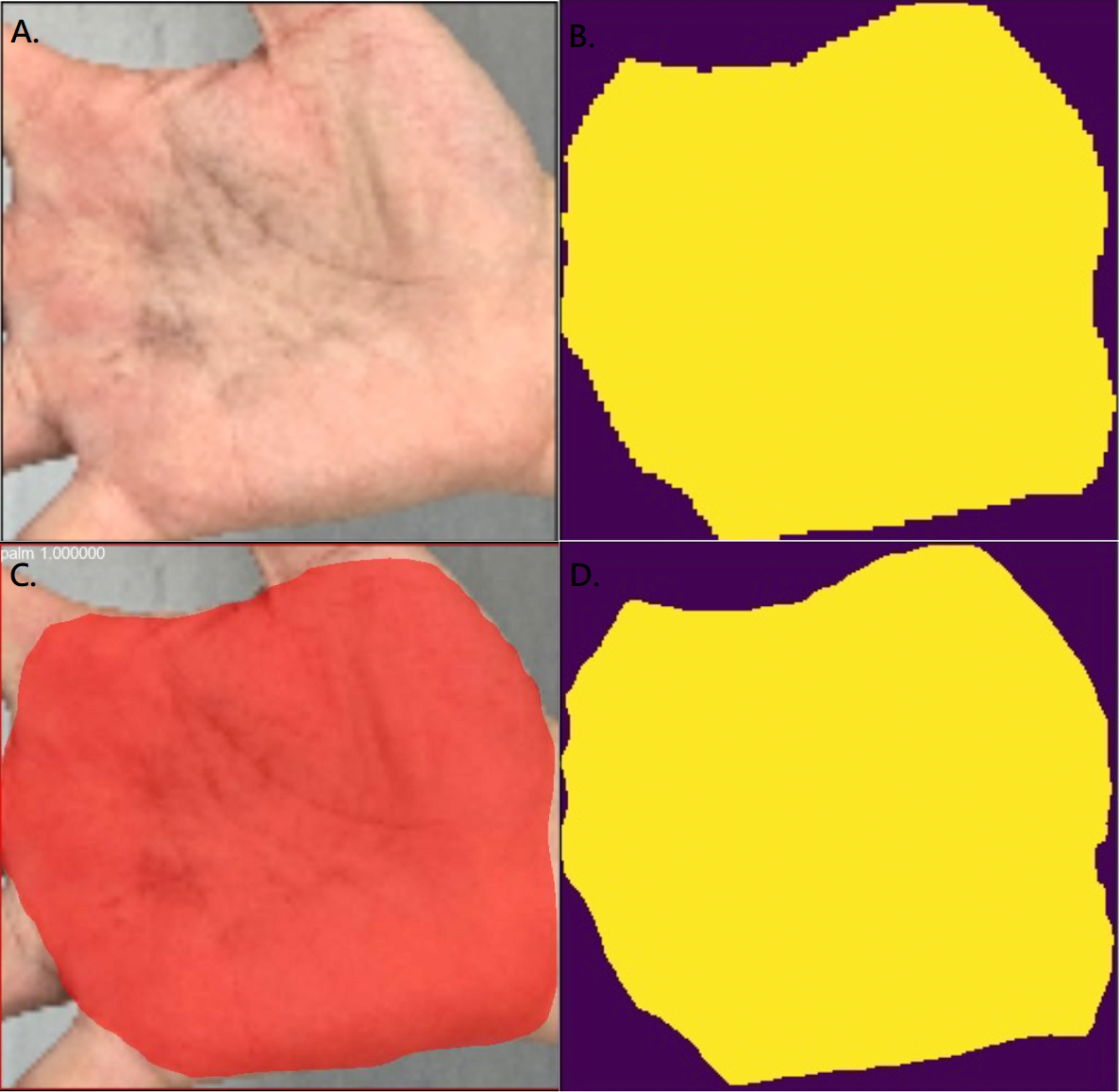

Supplement: Multimedia Appendix 2 [file medinform_v9i12e22798_app2.png]

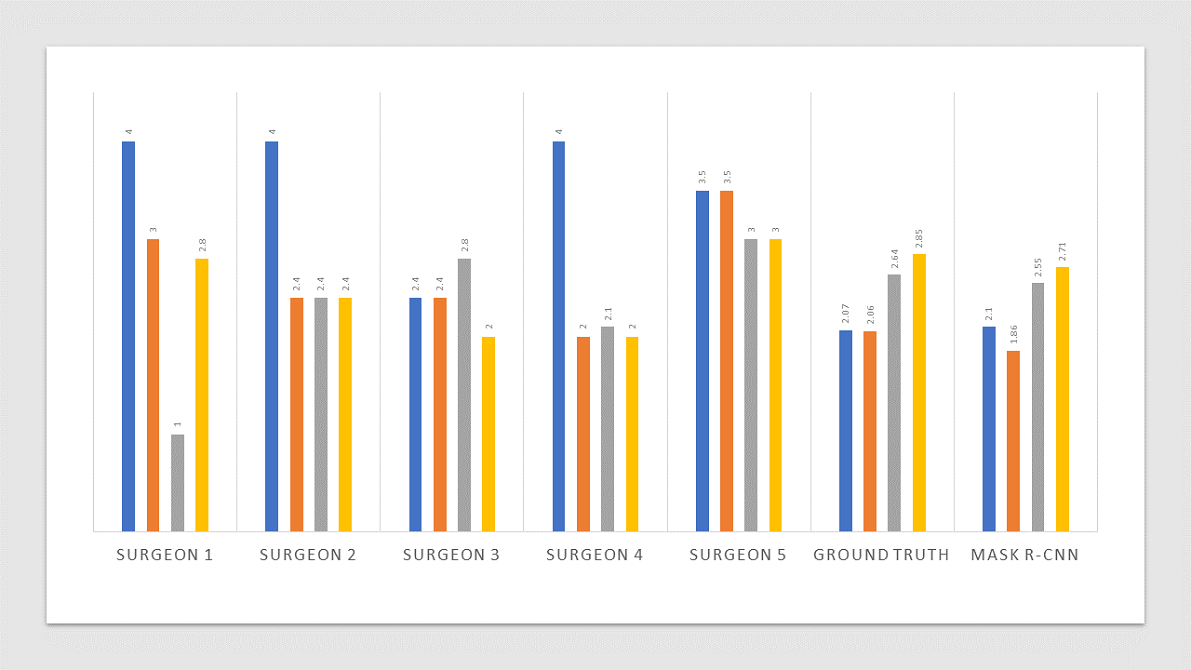

Supplement: Multimedia Appendix 3 [file medinform_v9i12e22798_app3.png]

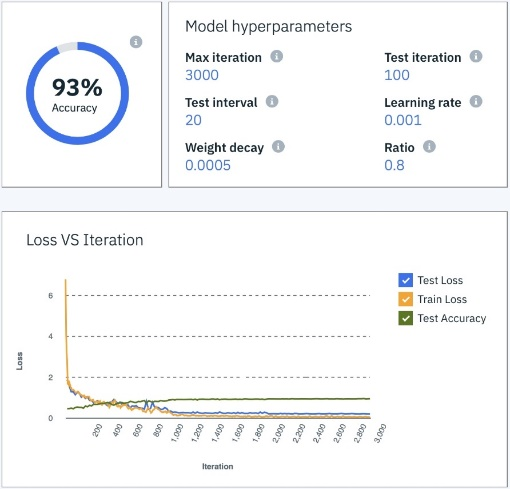

Supplement: Multimedia Appendix 4 [file medinform_v9i12e22798_app4.png]

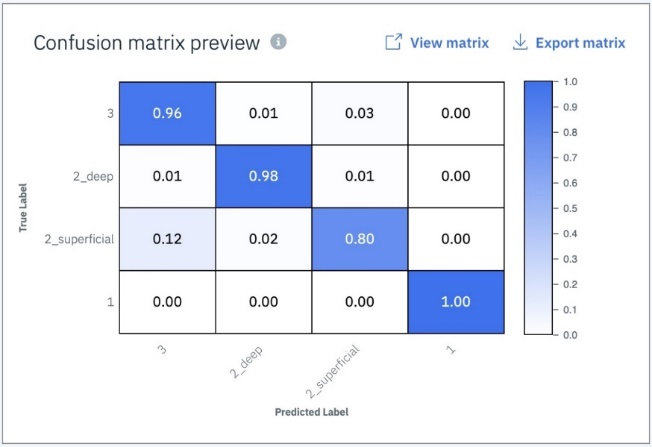

Supplement: Multimedia Appendix 5 [file medinform_v9i12e22798_app5.png]

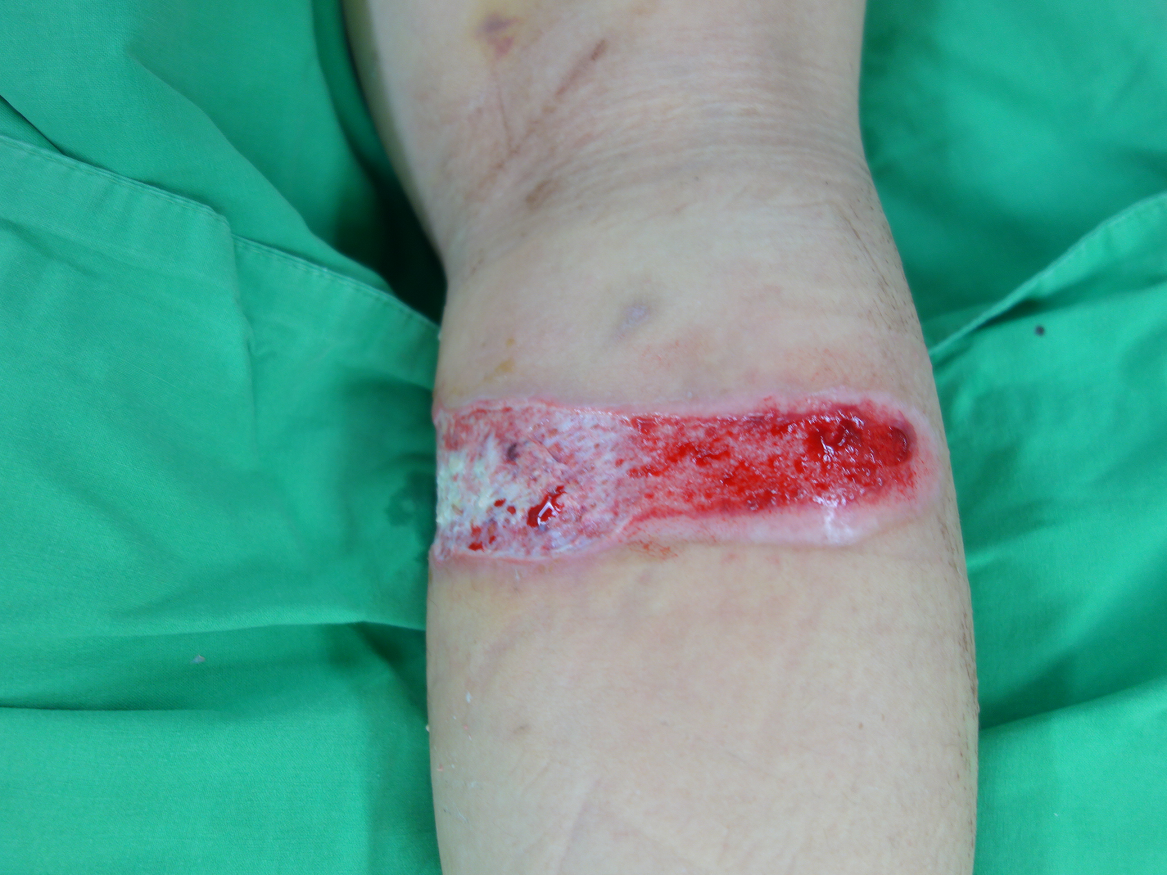

Supplement: Multimedia Appendix 6 [file medinform_v9i12e22798_app6.png]

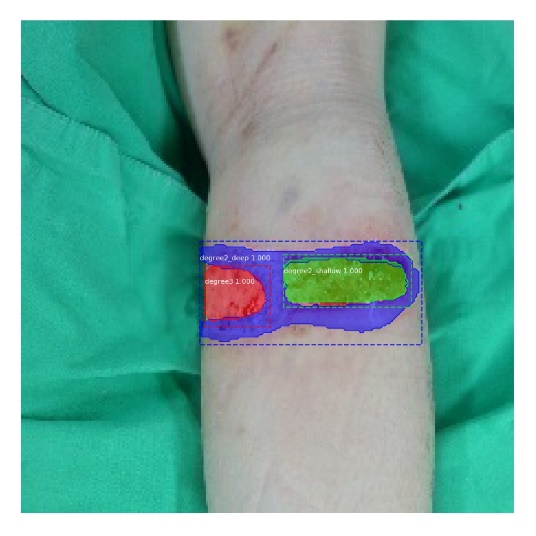

Supplement: Multimedia Appendix 7 [file medinform_v9i12e22798_app7.png]

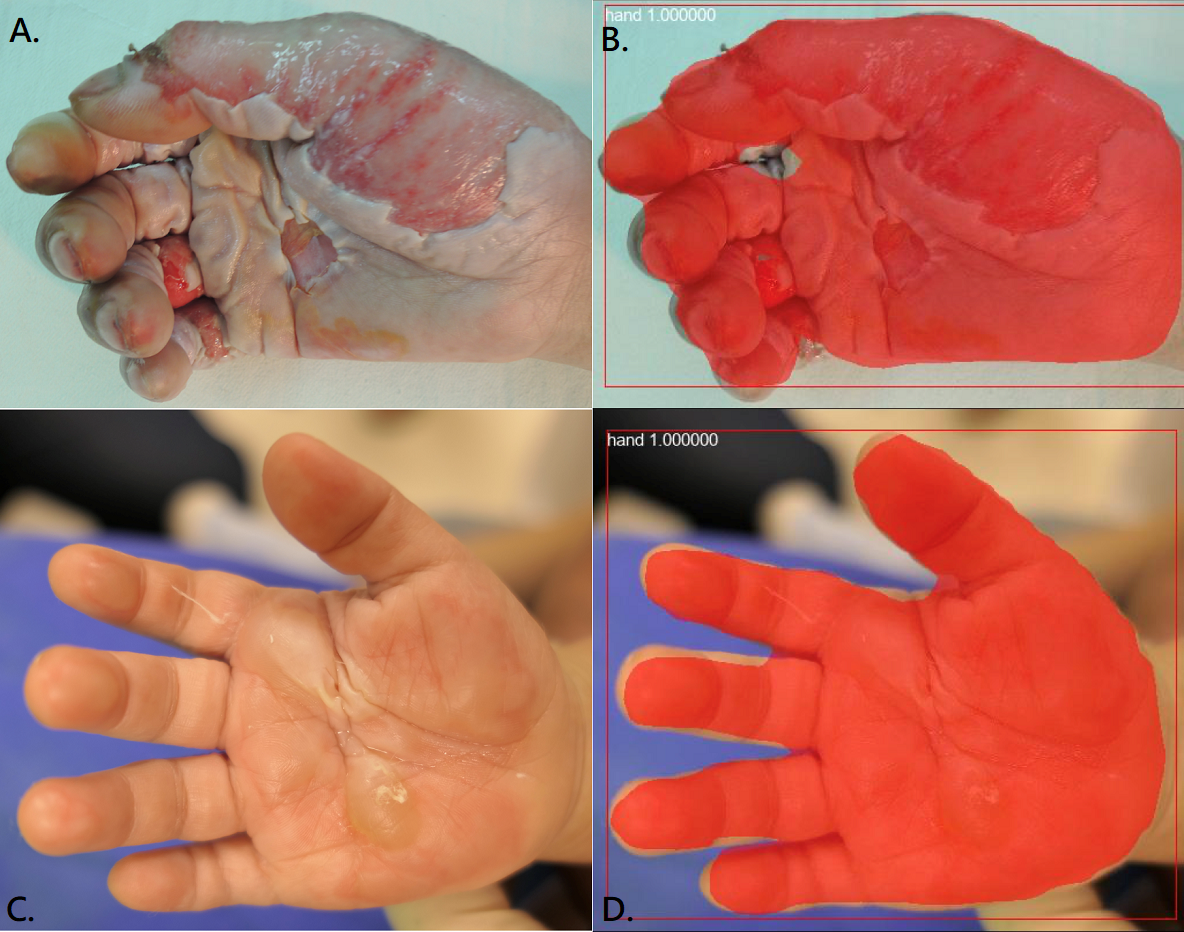

Supplement: Multimedia Appendix 8 [file medinform_v9i12e22798_app8.png]

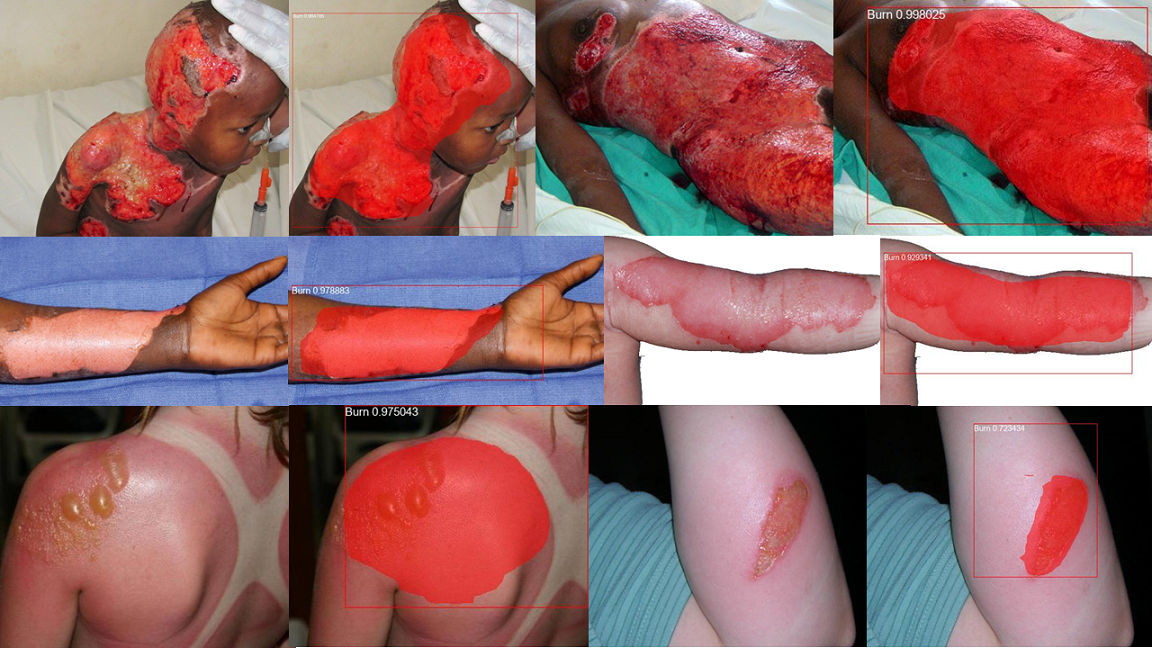

Supplement: Multimedia Appendix 9 [file medinform_v9i12e22798_app9.png]
